# Influence of Lymphangio (L), Vascular (V), and Perineural (Pn) Invasion on Recurrence and Survival of Resected Intrahepatic Cholangiocarcinoma

**DOI:** 10.3390/jcm10112426

**Published:** 2021-05-30

**Authors:** Fabian Bartsch, Lisa-Katharina Heuft, Janine Baumgart, Maria Hoppe-Lotichius, Rabea Margies, Tiemo S. Gerber, Friedrich Foerster, Arndt Weinmann, Beate K. Straub, Jens Mittler, Stefan Heinrich, Hauke Lang

**Affiliations:** 1 Department of General, Visceral and Transplant Surgery, University Medical Center of the Johannes Gutenberg-University Mainz, 55131 Mainz, Germany; fabian.bartsch@unimedizin-mainz.de (F.B.); lisa-katharina.heuft@unimedizin-mainz.de (L.-K.H.); janine.baumgart@unimedizin-mainz.de (J.B.); maria.hoppe-lotichius@unimedizin-mainz.de (M.H.-L.); rabea.margies@unimedizin-mainz.de (R.M.); jens.mittler@unimedizin-mainz.de (J.M.); Stefan.Heinrich@unimedizin-mainz.de (S.H.); 2 Department of Pathology, University Medical Center of the Johannes Gutenberg-University Mainz, 55131 Mainz, Germany; tiemo.gerber@unimedizin-mainz.de (T.S.G.); beate.straub@unimedizin-mainz.de (B.K.S.); 3 1st Department of Internal Medicine, Gastroenterology and Hepatology, University Medical Center of the Johannes Gutenberg-University Mainz, 55131 Mainz, Germany; friedrich.foerster@unimedizin-mainz.de (F.F.); arndt.weinmann@unimedizin-mainz.de (A.W.)

**Keywords:** intrahepatic cholangiocarcinoma, liver surgery, perineural invasion, lymphangioinvasion, vascular invasion, survival

## Abstract

(1) Background: Intrahepatic cholangiocarcinoma (ICC) is a rare malignancy. Besides tumor, nodal, and metastatic status, the UICC TNM classification describes further parameters such as lymphangio- (L0/L1), vascular (V0/V1/V2), and perineural invasion (Pn0/Pn1). The aim of this study was to analyze the influence of these parameters on recurrence and survival. (2) Methods: All surgical explorations for patients with ICC between January 2008 and June 2018 were collected and further analyzed in our institutional database. Statistical analyses focused on perineural, lymphangio-, and vascular invasion examined histologically and their influence on tumor recurrence and survival. (3) Results: Of 210 patients who underwent surgical exploration, 150 underwent curative-intended resection. Perineural invasion was present in 41, lymphangioinvasion in 21, and vascular invasion in 37 patients (V1 *n* = 34, V2 *n* = 3). Presence of P1, V+ and L1 was significantly associated with positivity of each other of these factors (*p* < 0.001, each). None of the three parameters showed direct influence on tumor recurrence in general, but perineural invasion influenced extrahepatic recurrence significantly (*p* = 0.019). Whereas lymphangio and vascular invasion was neither associated with overall nor recurrence-free survival, perineural invasion was significantly associated with a poor 1-, 3- and 5-year overall survival (OS) of 80%, 35%, and 23% for Pn0 versus 75%, 23%, and 0% for Pn1 (*p* = 0.027). Concerning recurrence-free survival (RFS), Pn0 showed a 1-, 3- and 5-year RFS of 42%, 18%, and 16% versus 28%, 11%, and 0% for Pn1, but no significance was reached (*p* = 0.091). (4) Conclusions: Whereas lymphangio- and vascular invasion showed no significant influence in several analyses, the presence of perineural invasion was associated with a significantly higher risk of extrahepatic tumor recurrence and worse overall survival.

## 1. Introduction

Intrahepatic cholangiocarcinoma (ICC) is a rare malignancy with poor prognosis. Surgical resection offers the only chance of cure. Due to a frequently late onset of symptoms, ICC is often diagnosed in an advanced stage. Therefore, extended resections with visceral and vascular resection and reconstruction are often necessary for complete tumor clearance. After resection, 5-year overall survival varies between 19–24% [1,2,3,4].

A wide variety of parameters have been included in single or multicentric analyses of survival. One of the most often identified predictors of poor survival is multifocality [5,6,7,8,9,10], followed by N-status [3,5,7,11]. Further factors such as R-status, UICC-stage, and tumor size have significant influence [1,2,7,12,13,14]. In addition to the tumor, nodal, and metastatic status, the UICC TNM classification describes further parameters such as lymphangioinvasion (L0/L1), vascular (V0/V1/V2), and perineural invasion (Pn0/Pn1) [15]. Whereas for L- and Pn-status, only absence (L0/Pn0) or invasion (L1/Pn1) exist as parameters, vascular invasion is further differentiated in no (V0), microscopic (V1), or macroscopic invasion (V2). The relevance of these parameters, especially of L- and V-status, has not been described in detail for ICC. Pn-status has been included in some studies on ICC, but only a few studies have focused on perineural invasion as a main parameter. Nakagohri and colleagues detected a significant influence of perineural invasion on 5-year overall survival in 40 patients with ICC [16]. Another publication by Shirai and colleagues focused on perineural invasion and contributed detailed insight into the associated factors [17]. They were able to show a significant influence of several parameters, such as tumor location and lymphatic invasion, on perineural invasion.

The aim of this manuscript was to analyze the influence of lymphangioinvasion (L-status), vascular invasion (V-status), and perineural invasion (Pn-status) on tumor recurrence, overall survival and recurrence-free survival in a large European single-center cohort of resected intrahepatic cholangiocarcinoma.

## 2. Materials and Methods

Data of all patients who underwent surgical exploration for ICC were included in a prospective institutional database. The observation period started in January 2008 and ended at the end of June 2018. Other malignancies, such as perihilar and distal cholangiocarcinoma and hepatocellular or gallbladder carcinoma, were excluded. Centrally located tumors with contact and/or infiltration of the liver hilum exceeding a diameter of 3 cm and an obvious origin of secondary or tertiary bile ducts (in preoperative imaging and/or histologically) were included as ICC.

All patients signed an informed consent form that data and follow-up may be collected anonymously and used for scientific analyses. According to the regulations of the federal state law (state hospital laws §36 and §37) and the independent ethics committee of Rheinland-Palatinate, no ethical approval was necessary for this study.

### 2.1. Preoperative Work-Up, Wurgical Procedures, and Follow-Up

For staging and operation planning, we preferred contrast-enhanced multiphasic computed tomography (CT) of the abdomen and thorax. A variety of patients presented with the initial diagnosis and imaging already made at referring centers or in an ambulant setting. We accepted externally produced CT scans or magnetic resonance imaging (MRI) if their quality was sufficient. We did not seek preoperative histological confirmation through biopsy if resection seemed technically possible. If metastatic disease from the gastrointestinal tract was questionable, gastroscopy and colonoscopy were performed to exclude another liver metastasis of another primary tumor.

All surgical explorations and resections were conducted by a team of experienced surgeons with special expertise in hepatobiliary surgery. The general course of action at our center is to perform even visceral or vascular resections and reconstructions to achieve complete tumor clearance, if reasonable. Lymphadenectomy is routinely performed aiming at least 6 harvested lymph nodes. Morbidity was classified according to the Clavien-Dindo classification [18].

Every 3 months after initial surgery for at least 2 years, we routinely conducted follow-up. Then, the interval was increased to 6 months, if reasonable. Preferable CT imaging (or MRI) was carried out at least every 6 months in turns with ultrasound examinations. For patients who were not able to undergo follow-up at our center, for example, due to logistic reasons, we stayed in contact with the treating physician to obtain all information needed.

### 2.2. Data Analysis

Data analysis focused on lymphangioinvasion (L0/1), vascular (V0/V1/V2), or perineural invasion (Pn0/Pn1). These parameters were examined and proven or excluded through histological/pathological study of the specimen and classified according to the 8^th^ edition of the TNM classification [15].

### 2.3. Statistical Analyses

Statistical analyses were performed with SPSS 23 (SPSS Inc., released 2014, IBM SPSS Statistics for Windows, Version 23.0, IBM Armonk, NY, USA: IBM Corp). For the analysis of categorical data, we used the Chi2 test in cross tabulation. Overall survival and recurrence-free survival analysis was conducted with the Kaplan–Meier method and log-rank test. A *p*-value of < 0.05 was considered significant. Multivariate analysis was performed using the Cox regression model. All analyses were intention-to-treat. Recurrence-free survival was classified after Punt et al. [19].

## 3. Results

In total, 210 patients with 150 resections were included in this study. Median age was 64.2 years (IQR 56.2–74.1; range 32.3–84.4) and gender was distributed almost equally female *n* = 102; male *n* = 108). Most patients were classified as ASA II (*n* = 91; American Society of Anesthesiologist’s classification) or III (*n* = 113) (ASA I *n* = 2, IV *n* = 4). Of the 210 patients, 14 underwent preoperative chemotherapy, of which 11 underwent resection.

In total, 60 ICC were unresectable due to peritoneal carcinomatosis (*n* = 23), multifocal tumor spread (*n* = 15), locally advanced infiltration (*n* = 11), or cirrhosis/small for size liver remnant/poor quality of liver parenchyma (*n* = 11).

### 3.1. Distribution of Lymphangio-, Vascular, and Perineural Invasion

The distribution of lymphangio-, vascular, and perineural invasion is shown in Table 1. In cross tabulation, positivity of the three different parameters is connected to each other. For all combinations, significant results were achieved (V0/V+ vs. L0/L1 *p* < 0.001; V0/V+ vs. Pn0/Pn1 *p* < 0.001; L0/L1 vs. Pn0/Pn1 *p* < 0.001).

### 3.2. Factors Associated with Perineural, Lymphangio-, and Vascular Invasion

Association of perineural, lymphangioinvasion, and vascular invasion with other histological and procedure related factors are shown in Table 1. N-status, L-status, V-status, major resection, and visceral invasion showed a significant correlation with perineural invasion. T-status, N-status, Pn-status, and V-status showed significant influence on lymphangioinvasion. Beneath Pn-status and L-status, vascular invasion was significantly higher in patients who also needed major resection.

There was no association of Pn-status (*p* = 0.532), L-status (*p* = 0.156), or V-status (V0/V1 + V2; *p* = 0.537) with postoperative morbidity (Clavien-Dindo classification grades 0-II vs. IIIa–V).

### 3.3. Overall Survival

**Figure 1:**
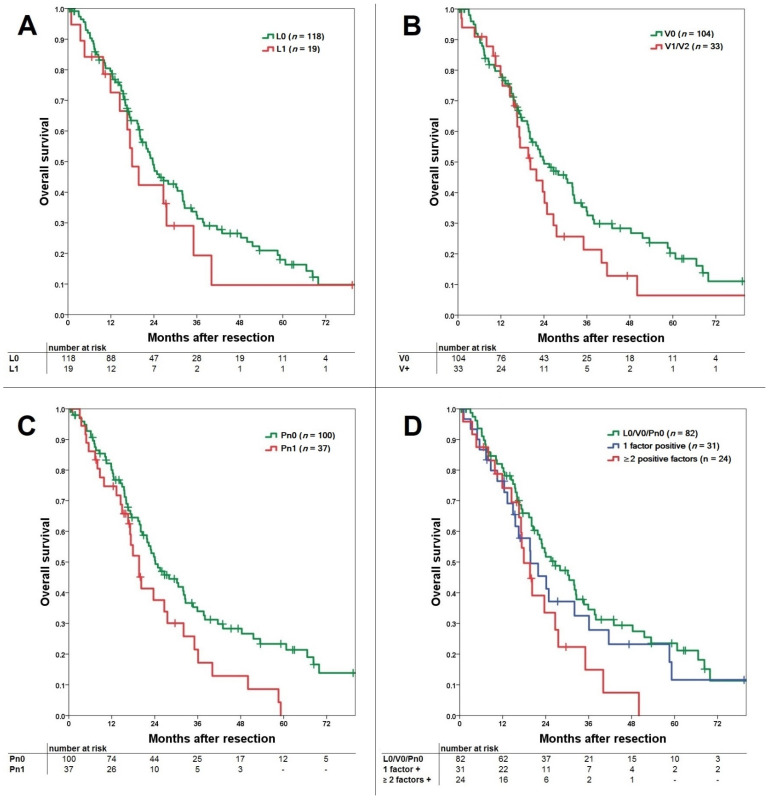
(**A**) Comparison of overall survival for lymphangioinvasion with comparable survival for the L0 and L1 group (*p* = 0.369). (**B**) Comparison of overall survival for vascular invasion with comparable survival for the V0 and V+ (V1 + V2) group (*p* = 0.149). (**C**) Comparison of overall survival for perineural invasion with significantly better survival for the Pn0 over the Pn1 group (*p* = 0.027). (**D**) Comparison of overall survival for L0/V0/Pn0 versus 1 positive factor and ≥2 positive factors groups (*p* = 0.091). In subgroup comparison the L0/V0/Pn0 was significantly better compared to the ≥2 positive factors group (*p* = 0.025), while 1 positive factor vs. ≥2 positive factors was comparable (*p* = 0.258).

Median follow-up of the resected patients was 19.6 months. For patients with lymphangioinvasion, the median OS was 24 months for L0 patients and 20.9 months for L1 patients (*p* = 0.369). OS was comparable with a consecutive 1-, 3-, and 5-year survival of 80%, 33%, and 18% for L0 versus 72%, 21% and 11% for L1, respectively (Figure 1A). For patients with vascular invasion (V0 vs. V1/V2), median OS was 25.2 months for V0 and 21 months for the V-positive group. OS was comparable with a consecutive 1-, 3-, and 5-year survival of 79%, 35%, and 21% for V0 versus 78%, 21%, and 6% for V-positive patients, respectively (*p* = 0.149; Figure 1B). For patients with perineural invasion, median OS was 25.5 months for Pn0 patients and 20.5 months for Pn1 patients. OS was significantly better for the Pn0 group with a consecutive 1-, 3-, and 5-year survival of 80%, 35%, and 23% versus 75%, 23%, and 0% for Pn1, respectively (*p* = 0.027; Figure 1C).

Comparing OS for L0/V0/Pn0 vs. one positive factor vs. ≥two positive factors, no significant difference was shown (*p* = 0.091; Figure 1D). OS for L0/V0/Pn0 was significantly better compared to ≥two positive factors (*p* = 0.025), while one positive vs. ≥two positive factors showed no significant difference (*p* = 0.258).

### 3.4. Recurrence-Free Survival

**Figure 2:**
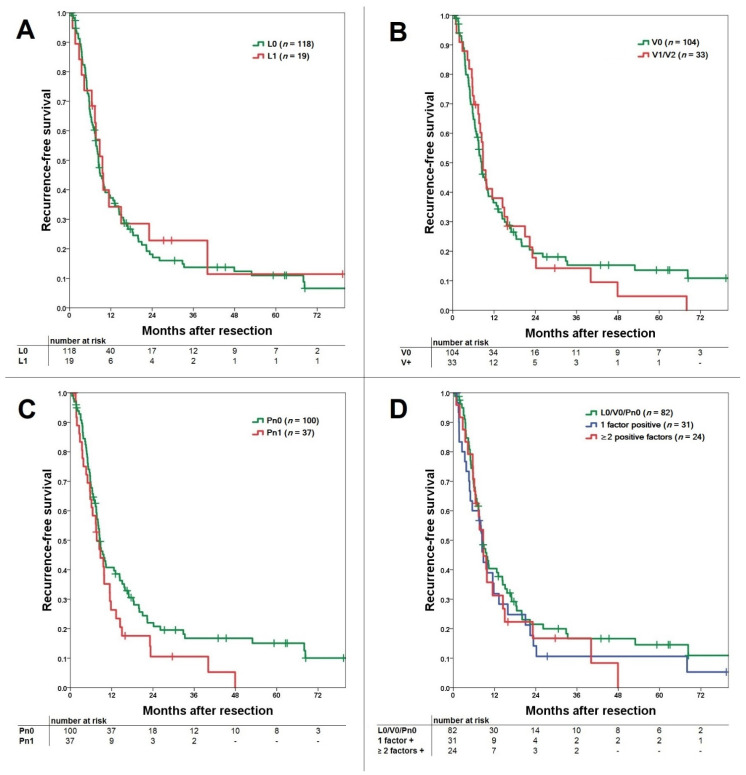
(**A**) Comparison of recurrence-free survival for lymphangioinvasion with comparable survival for the L0 and L1 group (*p* = 0.673). (**B**) Comparison of recurrence-free survival for vascular invasion with comparable survival for the V0 and V+ (V1/V2) group (*p* = 0.818). (**C**) Comparison of recurrence-free survival for perineural invasion with better survival for the Pn0 over the Pn1 group without reaching significance (*p* = 0.091). (**D**) Comparison of recurrence-free survival for L0/V0/Pn0 versus 1 positive factor and ≥2 positive factors groups (*p* = 0.525). In subgroup comparison, no difference can be shown for L0/V0/Pn0 vs. ≥2 positive factors (*p* = 0.414) and 1 positive factor vs. ≥2 positive factors (*p* = 0.999).

For lymphangioinvasion, the median RFS was 9.8 months for L0 patients and 9.3 months for L1 patients. RFS was comparable with a consecutive 1-, 3-, and 5-year survival of 39%, 15%, and 12% for L0 versus 35%, 23%, and 12% for L1, respectively (*p* = 0.673; Figure 2A). For vascular invasion, median RFS was 9.7 months for V0 and 9.8 months for V-positive patients. RFS (V0 vs. V+) was comparable with a consecutive 1-, 3-, and 5-year survival of 38%, 16%, and 14% for V0 patients versus 38%, 14%, and 5% for V1/V2 patients, respectively (*p* = 0.818; Figure 2B). For perineural invasion, median RFS was 10.3 months for Pn0 and 8.3 months for Pn1 patients. RFS showed a comparable RFS with a consecutive 1-, 3-, and 5-year survival of 42%, 18%, and 16% for Pn0 versus 28%, 11%, and 0% for Pn1, respectively (*p* = 0.091; Figure 2C).

Comparing RFS for L0/V0/Pn0 vs. one positive factor vs. ≥two positive factors, no significant difference can be shown (*p* = 0.525; Figure 2D). Even subgroup analysis shows that L0/V0/Pn0 was comparable to ≥2 positive factors (*p* = 0.414), as well as one positive versus ≥2 positive factors (*p* = 0.999).

### 3.5. Influence on Tumor Recurrence

Tumor recurrence occurred in 97 patients. Tumor recurrence was most commonly intrahepatic (*n* = 42; 43.3%), followed by combined intra- and extrahepatic recurrence (*n* = 30; 30.9%) and only extrahepatic recurrence (*n* = 25; 25.8%). Tumor recurrence was neither influenced by L-status (*p* = 0.163), V-status (*p* = 0.574), nor Pn-status (*p* = 0.153). No influence could be detected (*p* = 0.478), even if all factors were negative (L0/V0/Pn0), one factor was positive, or ≥two factors were positive regarding the occurrence of tumor recurrence. Localization of recurrence was significantly influenced by Pn-status with predominant extrahepatic recurrence (*p* = 0.019). L-status (*p* = 0.875), V-status (*p* = 0.627) as well as L0/V0/Pn0 versus one positive factor or ≥two positive factors (*p* = 0.256) had no influence.

### 3.6. Multivariate Analysis

Using univariate analysis, several parameters were tested. Some factors showed to be significantly associated with OS and RFS and were further included in multivariate cox regression analysis (Table 2). For OS, N-stage and tumor size were independent predictors. For RFS, M-stage, tumor size, and multifocality were independent predictors.

## 4. Discussion

The influence of perineural invasion (Pn), and especially lymphangioinvasion (L) and vascular invasion (V), has only seldom been explicitly analyzed in patients who underwent resection for ICC. We showed that perineural invasion is significantly associated with worse overall survival, contrary to lymphangioinvasion or vascular invasion. Whereas the incidence of tumor recurrence was not directly associated with Pn-, L-, or V-status, perineural invasion affected extrahepatic recurrence significantly.

The TNM classification provided by the Union for International Cancer Control (UICC) is a generally accepted and utilized instrument to classify tumor extension. The eighth edition is the latest version deployed worldwide, and many changes were applied for ICC in comparison to the seventh edition [15,20]. Compared to the seventh edition, an improvement toward a more precise classification of ICC can be seen in the eighth edition [21]. However, the system is not free of critics. Some authors have developed their own modifications to postulate better discrimination of survival or proposed adjustments for the latest editions [5,22,23,24]. Next to the classic T-, N-, and M-status which vary between different tumor entities, the TNM classification adds standardized factors for perineural invasion, lymphangioinvasion, and vascular invasion. Whereas perineural invasion has seldomly been addressed as the main focus [17,25], it is often included in the evaluation of prognostic factors, and variations have been shown to significantly influence survival [2,3,12,26] or not significantly [6,27]. In contrast, lymphangioinvasion and vascular invasion have been mentioned only scarcely [1,12].

We showed that either Pn1, V1/V2, or L1 were significantly associated with positivity of any other of these parameters (see Table 1 and Table 2). Data regarding this correlation are scarce, as only Shirai and colleagues showed a significant connection between perineural and lymphangioinvasion in an analysis of 59 resected patients [17]. An association between vascular and perineural invasion could not be shown. This might be related to the lower number of patients included. A correlation between these factors is reasonable because all factors are signs of tumor aggressiveness.

Factors associated with perineural invasion were N-status, major resection, and visceral infiltration, in addition to the already mentioned connection to V- and L-status. A comparable analysis by Zhang and colleagues in a Chinese cohort of 134 resected ICC showed Hepatitis B surface antigen and CA 19–9 as influencing parameters for perineural invasion positivity [25]. The visceral invasion was tested as well but showed no significance. Shirai and colleagues revealed that tumor location, tumor configuration, nodal involvement, extrahepatic portal vein invasion, histologic grade, and lymphangioinvasion were associated with perineural invasion [17]. The findings of Shirai et al. correspond with our results because most parameters are associated with advanced tumor stage. The data from China, however, is contradictory, because many comparable parameters were tested, but did not show any influence.

Lymphangioinvasion was associated with T-status and N-status, while vascular invasion correlated solely with major resections. The findings for lymphangioinvasion are comprehensible and not surprising, especially the correlation with the N-status. However, it is unexpected that vascular invasion is solely found in patients with major resection. Of course, larger segmental extension is associated with advanced tumor growth. Nevertheless, other parameters connected to tumor aggressiveness, such as T- or N-status, did not significantly correlate with vascular invasion. The comparable small number of V1/V2 patients may have influenced this result. We found no other studies which performed comparable analyses to discuss these results.

Tumor recurrence of ICC is common, and its absence is clearly the most important factor regarding long-term survival. Factors influencing tumor recurrence are well analyzed and numerous, including preoperative CA19–9, tumor size, multifocality, visceral infiltration, vascular infiltration, perineural invasion, T-status, grading, and lymph node metastasis, particularly N-status [28,29,30]. In our analysis, neither perineural, vascular, nor lymphangioinvasion had a significant influence on tumor recurrence. Perineural invasion was significantly associated only with the appearance of extrahepatic recurrence. This corresponds with a comparable analysis by Spolverato and colleagues on a large multicentric cohort which demonstrated significant influence on extrahepatic recurrence as well [30]. In principle, one would expect that all three parameters (Pn, L, and V) have the potential to influence tumor recurrence. Whereas other studies have focused on perineural invasion [29] and vascular invasion [28,30], lymphangioinvasion has been scarcely studied.

Perineural invasion was significantly associated with overall survival and showed at least a slightly better recurrence-free survival of Pn0 over Pn1, but without reaching significance. In contrast, lymphangioinvasion and vascular invasion showed only a decently better survival for the L0 or V0 groups for overall survival and nearly no difference for recurrence-free survival. A recent study from China analyzed 134 patients who underwent curative-intended resection for ICC. The perineural invasion had a significant influence on both overall and recurrence-free survival (*p* < 0.001, each) [25]. Vascular invasion showed no significant influence, and lymphangioinvasion was not analyzed or represented. In a large international multicenter study by Bagante and colleagues with 679 patients, the presence of perineural and microvascular invasion was associated with worse long-term survival, but macrovascular (V2) and lymphatic invasion were not tested [31]. In a systematic review and meta-analysis, Mavros and colleagues analyzed 57 studies [32], finding that recurrence-free survival was influenced by perineural invasion in 2 of 5, by microvascular invasion in 1 of 3, and by major vascular invasion in 5 of 8 studies. For overall survival, vascular invasion was associated in 13 of 19, microvascular invasion in 4 of 7, and perineural invasion in 7 of 12 studies. Lymphangioinvasion was not mentioned. Considering that 57 studies were included, it is obvious that Pn-, L-, and V-status are not often addressed. Only vascular invasion for overall survival was included rather often.

In multivariate analysis, only perineural invasion was included for OS and RFS. However, perineural invasion was not an independent predictor of survival for OS or RFS. For RFS and OS, tumor size, N-stage, M-stage, and multifocality showed significance according to the literature [3,5,7,10,11,14].

Our study has some limitations. The retrospective character reduces the validity. Furthermore, due to the limited number of patients within the subgroups, analyses regarding lymphangioinvasion and vascular invasion might be especially underpowered to reach significant results. Additional studies with a higher number of patients are necessary to prove our findings, most preferably with a prospective design.

## 5. Conclusions

The presence of perineural invasion led to a significantly higher risk of extrahepatic tumor recurrence and showed worse overall survival. Lymphangioinvasion and vascular invasion showed no significant influence in several analyses.

## Figures and Tables

**Table 1:** Factors associated with perineural, lymphatic, and venous infiltration.

	Pn1	Pn0	*p*	L1	L0	*p*	V+	V0	*p*
	*n* = 41	*n* = 109		*n* = 21	*n* = 129		*n* = 27	*n* = 113	
T-status			0.128			**0.046**			0.253
T1 + T2	27	85		12	100		25	87	
T3 + T4	14	24		9	29		12	26	
N-status *			**0.019**			**0.010**			0.121
N0	20	70		8	82		20	70	
N1	18	25		11	32		15	28	
M-status			0.723			0.085			0.861
M0	39	101		18	123		35	106	
M1	2	7		3	6		2	7	
Pn-status			-			**<0.001**			**<0.001**
Pn0	-	-		9	100		17	92	
Pn1	-	-		12	29		20	21	
L-status			**<0.001**			-			**<0.001**
L0	29	100		-	-		22	92	
L1	12	9		-	-		13	6	
V-status			**<0.001**			**<0.001**			-
V0	21	92		6	92		-	-	
V1 + V2	20	17		13	22		-	-	
Grading			0.162			0.124			0.212
G1 + G2	22	72		10	84		20	74	
G3 + G4	19	37		11	45		17	39	
R-status			0.320			0.641			0.696
R0	34	97		19	112		33	98	
R1	7	12		2	17		4	15	
Multifocality			0.820			0.191			0.788
Yes	12	34		9	37		12	34	
solitary	29	75		12	92		25	79	
≥major resection			**0.004**			0.504			**0.012**
Yes	36	69		16	89		32	73	
No	5	40		5	40		5	40	
Extended resection			0.410			0.299			0.174
Yes	26	61		10	77		25	62	
No	15	48		11	52		12	51	
Visceral infiltration			**0.015**			0.406			0.250
Yes	10	10		4	16		7	13	
No	31	99		17	113		30	100	

Significant *p*-values in bold; * 17 patients with Nx were excluded from this analysis.

**Table 2:** Univariate and multivariate analysis.

		Kaplan Meier		Multivariate Cox Regression					
		OS	RFS	OS			RFS		
				HR	95% CI	*p*-Value	HR	95% CI	*p*-Value
Age	<65/>65	0.073	0.175	1.375	0.892–2.117	0.149			
Gender	Woman/Man	0.283	0.472						
Extended res.	yes/no	**0.009**	**0.024**	0.637	0.400–1.015	0.058	0.699	0.468–1.043	0.079
Tumor size	≤5cm/>5 cm	0.108	**0.008**						
	≤ 10 cm/>10 cm	**0.014**	**0.002**	**1.678**	**1.012–2.780**	**0.045**	**1.697**	**1.090–2.641**	**0.019**
Multifocality	yes / no	0.262	**0.014**				**1.540**	**1.029–2.304**	**0.036**
T-stage	T1 + T2/T3 + T4	0.102	0.347						
N-stage	N0/N+ / NX	**0.016**	0.085	**1.910**	**1.190–3.065**	**0.007**	1.084	0.891–1.318	0.420
V-stage	V0/V1 + V2	0.149	0.818						
L-stage	L0/L1	0.369	0.673						
Pn-stage	Pn0/Pn1	**0.027**	0.091	1.395	0.864–2.252	0.173	1.376	0.907–2.088	0.133
M-stage	M0/M1	0.125	**0.002**				**3.133**	**1.384–7.003**	**0.006**
R-stage	R0/R1	0.655	0.254						
Grading	G1 + G2/G3 + G4	0.347	0.535						
UICC stage	Stage I + II/III + IV	**0.035**	0.155	1.334	0.661–2.691	0.421			

Perioperative deaths were excluded for statistical analyses; significant parameters are bold; parameters with *p* < 0.1 were included in multivariate analyses (underlined); OS = overall survival, RFS = recurrence-free survival, HR = hazard ratio, 95% CI = 95% confidence interval.

## Data Availability

The datasets used and analyzed during the current study are available from the corresponding author on reasonable request.
